# Dr. Abby Janet Seymour

**Published:** 1895-11

**Authors:** 


Dr. Abby Janet Seymour, the most prominent woman homeopathic physician in Buffalo, was killed by a railway train on the New York Central tracks, near the water works station, Thursday evening, October 17, 1895. It is supposed that she was struck by a train and instantly killed, but just how the accident occurred no one can tell, as there were no witnesses to it. Dr. Seymour had been in poor health for some time and was about forty-two years old at the time of her death. She was the daughter of the late Erastus Seymour, formerly one of Buffalo's leading citizens. She was educated at Mrs. Reed's school in New York and had lately taken a post-graduate medical course at Chicago. Dr. Seymour was an artist of considerable note and was a woman of diversified talent. Her loss will be deeply felt by a large circle of friends.

